# 0734. Early changes in heart rate predict long-term survival in a rodent model of sepsis

**DOI:** 10.1186/2197-425X-2-S1-P56

**Published:** 2014-09-26

**Authors:** A Rudiger, M Arrigo, T Hauffe, DR Spahn, D Bettex

**Affiliations:** Institute of Anesthesiology, University Hospital Zurich, Zurich, Switzerland; Clinic for Cardiology, University Hospital Zurich, Zurich, Switzerland

## Introduction

In sepsis, early prognostication of outcome would be helpful both for the detection of underlying mechanisms (adaptive vs. maladaptive) and the development of novel treatments for potential non-survivors.

## Objectives

We tested whether telemetry derived heart rate (HR) changes during early sepsis could prognosticate outcome in a long-term rat model of fecal peritonitis.

## Methods

Male Wistar rats were instrumented with telemetry for continuous HR and temperature recording. Abdominal sepsis was induced by intraperitoneal injection of fecal slurry. A jugular catheter allowed the intravenous administration of opioids (nalbuphin 1mg/kg/h), antibiotics (rocephin 30mg/kg at 4 and 24h) and crystalloids (bolus of 20ml/kg at 4h, followed by an infusion of 10ml/kg/h, halved after 8 and 24h). The awake animals were followed for 48 hours.

## Results

Septic animals became lethargic and febrile. Mortality was 30%, and all deaths occurred between 4 and 24 hours. Postmortem examination showed variable degrees of peritonitis and purulent ascites. Rats surviving 24 hours showed clinical signs of recovery.

**Table 1:** Heart rate (bpm) in survivors and non-survivors:

	Survivors (n=14)	Non-survivors (n=6)	p-value
At BL	405 (329-471)	394 (296-439)	0.31
4 h after septic insult	389 (318-440)	452 (349-494)	0.11
6 hours post septic insult	416 (331-494)	500 (418-594)	0.02
Change BL to 4 hours	-10 (-87-49)	61 (-52-85)	<0.01

Values are median (range); BL = baseline

ROC analysis revealed that the change in HR between baseline and 4h was a good prognosticator (AUC 0.84, 95% CI 0.56-1.00; p=0.03). An increase in HR of ≥50 bpm between baseline and 4 hours separated survivors and non-survivors with a sensitivity and specificity of 80% and 100%, respectively.

## Conclusions

In this clinically relevant rat model of abdominal sepsis changes in HR as early as 4 hours after the septic insult predicted outcome with good sensitivity and excellent specificity. This will allow future investigation of adaptive changes in potential survivors and mechanisms of death in potential non-survivors. In addition, novel sepsis-treatments (e.g. beta-blockers) can be tested in regards of their beneficial and harmful effects in potential non-survivors and survivors.
